# The influence of surface–groundwater interactions on nutrient dynamics in urban in-channel treatment systems

**DOI:** 10.1007/s10661-024-13459-4

**Published:** 2024-12-11

**Authors:** Fabio C. Silveira, Thomas A. Cochrane, Ricardo Bello-Mendoza, Frances Charters

**Affiliations:** Department of Civil and Natural Resources Engineering, University of Canterbury, Private Bag 4800, 8140 Christchurch, New Zealand

**Keywords:** Nitrate, Phosphate, Ammonia, Nutrients, Seepage, Drainage, Water quality monitoring

## Abstract

In-channel water treatment systems remove excess nutrients through biological, chemical, and physical processes associated with the hyporheic zone. However, the impact of surface and groundwater interactions on these treatment processes is poorly understood. This research aims to assess the influence of varying groundwater conditions (neutral, drainage water, and groundwater seepage) and different bed sediment hydraulic conductivities on nitrogen and phosphorus dynamics in in-channel treatment systems. A flume containing bed sediment was used to study changes in surface water quality under different groundwater and bed sediment conditions. Compared to inlet and outlet concentrations, dissolved reactive phosphorus (DRP) and ammoniacal nitrogen (NH_4_-N) levels in the surface water increased by 11–65% and 10–51%, respectively, while nitrate (NO_3_-N) concentrations decreased by 11% under groundwater seepage conditions. The increase in NH_4_-N was due to ammonification, while the decrease in NO_3_-N was due to denitrification and mixing and dilution with the groundwater. The upward groundwater flux through the bed sediment transported both DRP and NH_4_-N into the surface water. Low hydraulic (LH) conductivity sediment led to greater changes in nutrient concentration than high hydraulic (HH) conductivity sediment (DRP increased by 65% and NH_4_-N by 51% for LH, compared to 11% and 10% for HH, respectively). However, HH conductivity sediment led to greater variations in pH and Eh values. The findings could assist the design and monitoring of in-channel treatment systems where groundwater and surface water interact.

**Supplementary Information:**

The online version contains supplementary material available at 10.1007/s10661-024-13459-4.

## Introduction

Elevated nutrient levels in rural and urban waterways lead to eutrophication and degrade the health of aquatic ecosystems. Nutrient dynamics in waterways are influenced by the input of nitrogen and phosphorus from surface runoff, by infiltration and accumulation over time in the hyporheic zone, where shallow groundwater mixes with surface water around the streambed interface (Pescimoro et al., 2019), and by groundwater seepage. Surface–groundwater interactions in the hyporheic zone can change nutrient fluxes in the saturated sediment and in surface flows due to the impact of physical, chemical, and microbiological factors (i.e., particle size, sediment composition, microbiological species composition, hydrodynamic and hydraulic pressure gradients, and others). Residence time in the hyporheic zone, pore clogging due to fine sediments, carbon availability, diurnal and seasonal temperature variation, and seasonal variation of stratified groundwater contribution impact nutrient dynamics and availability and, subsequently, influence the aquatic ecology (Bohlke et al., 2007; Marçais et al., 2022; McCormack et al., 2016; Munz et al., 2017; Sunjidmaa et al., 2022).

Oxidized nitrogen removal in the water column of waterways primarily occurs through the denitrification process at the water–sediment boundary, driven by factors such as porosity, carbon content, oxygen levels, temperature, and residence time (Hampton et al., 2020; Munz et al., 2019). Among these, residence times within the hyporheic zone have been identified as the main driver of biochemical reactions (Zarnetske et al., 2011). A longer resident time increases the likelihood and extent of these reactions (Boano et al., 2014). In small streams, the large contact area between sediment and water flow area increases the chance of reactions to occur (Anderson et al., 2005).

Phosphorus retention in waterways is primarily achieved through adsorption to streambed sediments in the hyporheic zone and plant uptake (Jin et al., 2022a; Withers & Jarvie, 2008). In acid soils, aluminum (Al) and iron (Fe) are the key sorbents for phosphorus (Reddy & DeLaune, 2008). Nevertheless, phosphorus bonded to iron oxides in the bed sediments can be mobilized when conditions become anaerobic and/or anoxic (Forsmann & Kjaergaard, 2014; House & Denison, 2002).

In-channel treatment systems are commonly defined as treatment systems built in line with streams, drainage channels, and other waterways with the goal of removing contaminants (in this case nutrients) through biological, chemical, and physical processes by enhancing the natural treatment capabilities of the waterway itself (Moazzem et al., 2024; Walsh et al., 2016). Examples of these are engineered urban drainage streams (or waterways), constructed wetlands, and vegetated swales, where stormwater is not only conducted through them, but is also treated through interactions with soils and vegetation and through drainage/seepage (Chen et al., 2013; Kabenge et al., 2018; Leroy et al., 2016). However, the impact of surface and groundwater interactions on these treatment processes is poorly understood, especially with ongoing changes in surface and groundwater flow regimes as impacted by drainage and seepage (i.e., spring-fed waterways or other groundwater contributions) (Mangangka et al., 2016; Webster-Brown & Barr, 2016).

Experimental flumes can be used to isolate physical, chemical, and biological processes and to observe them in detail. Simplified flume experiments, like annular and linear flumes, have proved useful in understanding, modeling, and predicting processes between bed sediment and water column, such as advection-driven transport, production and removal of nutrients, resuspension, colloidal particles exchange, and others (Blom et al., 2003; Clark et al., 2019; Eylers et al., 1995; Harvey et al., 2011; Huang et al., 2015; Postma et al., 2008). For example, flume experiments have shown that nutrients bound to colloidal clay particles were trapped in the bed sediment at high surface water concentrations and released under low clay concentrations (Packman & Brooks, 1995). Furthermore, the release and entrapment of pore water due to sediment turnover were found to contribute to surface–groundwater water exchange under baseflow conditions, increasing its concentration in the surface water (Mutz & Rohde, 2003). Similarly, a study of phosphorus mobility triggered by sediment resuspension in a flume experiment with a recirculation system identified a strong relationship between phosphorus concentration in the surface water and sediment, greater concentrations in the bed sediment lead to great concentration in the surface water (Huang et al., 2015).

Field and modeling studies have shown that sediment (soil) permeability and surface water velocity through the sediment can increase nutrient supply and change the residence time of water within the streambed (Bardini et al., 2012). Furthermore, water flow gradients within ground sediments could lead to the movement of dissolved phosphorus (Withers & Jarvie, 2008) and the leaching of nutrients into the groundwater under drainage water conditions (Scott & Hanson, 2013; Yoder, 2014).

However, despite these findings of nutrient dynamics within the water column, bed sediment, and groundwater, significant research gaps remain. There is still a need to better understand nutrient transport and transformation under different flow conditions and bed sediment composition (e.g., gravel, sand, clay, organic matter) with different hydraulic conductivity and groundwater influence (neutral, drainage water, and groundwater seepage conditions). Current studies have not fully explored how these factors interact to affect nutrient dynamics in in-channel treatment systems, especially considering ongoing changes in surface and groundwater flow regimes seasonal variations and/or climate change, and modifications in waterways through geomorphic processes (between flow, sediment, and vegetation) and engineering interventions for stream restoration and improving water quality (Hatt et al., 2007; Hester & Doyle, 2008; Suddick et al., 2013; Vicente-Serrano et al., 2020; Vietz et al., 2016; Vietz et al., 2015). Moreover, quantifying nutrient transport and transformations under groundwater neutral, drainage, or seepage conditions and varying bed sediment hydraulic conductivity in in-channel treatment systems is necessary to understand the removal of nutrients via physical, chemical, and biological processes. It is important to note that although vegetation and bedforms are important aspects of in-channel treatment systems, in this study, we focus on flat-bed sediment interactions and on the surface/groundwater interactions through drainage and seepage. This understanding is crucial for guiding stream management decisions, designing effective in-channel treatment systems, and ultimately improving water quality in waterways.

This research thus aims to investigate nutrient dynamics (nitrogen and phosphorus) in a flume simulating an in-channel treatment system under different surface-water and ground-water interactions (neutral, drainage water, and groundwater seepage conditions) and bed sediment with low and high hydraulic conductivity. It is hypothesized that the surface–groundwater interactions and bed sediment properties influence the levels and form of nutrients, and their in-channel mobility. A better understanding of these processes could help the sustainable management of groundwater and surface water resources.

## Methodology

Column leaching tests, followed by flume experiments, were conducted to address the aims of the study. Contaminated bed sediments were sourced locally and mixed with coarse sand to enhance conductivity for flume and benchtop experiments. Synthetic stormwater was used in all experiments, with contaminant concentrations derived from observed surface-water quality from a local stream.

### Bed sediment

The bed sediment, used in all experiments, was sourced from Wigram Retention Basin (WRB) in Christchurch, New Zealand, a 30-year-old wet pond with a history of nutrient contamination (Black, 2018; Moores et al., 2009; Silveira et al., 2022). The WRB receives surface runoff from Haytons Stream, a groundwater-fed urban stream that receives stormwater runoff and direct discharges from a mixed industrial-residential catchment, including a fertilizer factory (Silveira, 2017). WRB sediment’s pH was 6.0, Olsen phosphorus concentration 37 mg/L (35.2 mg/kg soil based on volume weight 1.05 g/mL), organic matter 5.5%, total carbon 3.2%, and C/N ratio 13.3 (see Supplementary Information Figure A1, Table A1 and A2). Collected sediment was dried in a temperature-controlled room at 30 °C and 25% air humidity for 7 days, then sieved on a steel mesh with a 32-mm aperture. Silica sand was supplied by Commercial Minerals Ltd, Auckland, New Zealand (medium to coarse sand with D60 = 0.45 mm) (Chambers, 1999) and was then mixed with WRB’s bed sediment to enhance hydraulic conductivity. Two mixtures of bed media were prepared consisting of 40% sediment + 60% sand and 75% sediment + 25% sand.

### Synthetic stormwater

The synthetic stormwater (SSW) used in the experiments was prepared using potassium nitrate (KNO_3_), ammonium chloride (NH_4_Cl), and potassium dihydrogen phosphate (KH_2_PO_4_). It was then diluted with water to reach target concentrations of 0.4 mg/L of nitrate nitrogen (NO_3_-N) and 0.2 mg/L of both ammoniacal nitrogen (NH_4_-N) and dissolved reactive phosphorus (DRP). These concentrations simulated the observed in-channel water quality in Haytons Stream (CCC, 2022), while providing a consistent feed water quality between all experiments (see Table A3).

### Column leaching test methodology

Column leaching tests were conducted using 10-cm internal diameter and 30-cm-tall acrylic cylinders. Each column had 14 cm of gravel at the bottom topped with 4 or 5 cm of sediment mix. These tests were initially performed to assess the hydraulic conductivity of the sediment mixes with 50% WRB sediment and 50% sand and 20% WRB sediment and 80% silica sand, and to inform the selection of the blended sediment composition for the flume experiments. Water was applied to the top of columns with a static hydraulic head of around 2 cm, maintained by a lifting table that adjusted the height to achieve a target flow of 6 mL/min, as this represented the equivalent scaled-down flow of the subsequent flume experiment (Table A4; Table A5). Water flow was measured by collecting a known volume of water in a volumetric cylinder and using a chronometer to record the time taken. Residence time was calculated by dividing the volume of water in a system by the flow rate.

**Fig. 1:**
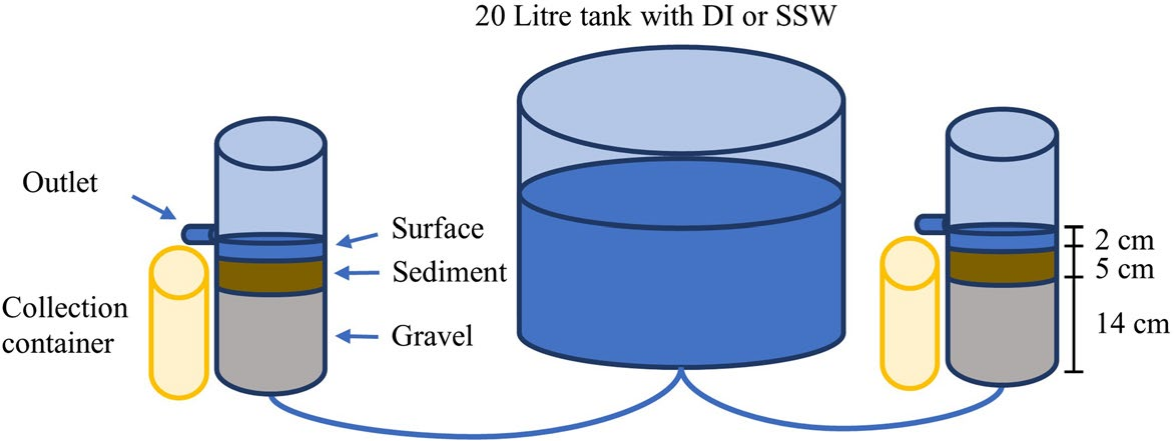
Schematic view of the column leaching test

Subsequent tests were conducted to determine changes in nutrient concentrations in the water column under groundwater seepage conditions using both deionized water (DI—low electrical conductivity) and synthetic stormwater (SSW, selected to have a low (DI)- and high electrical conductivity). The tests were conducted in duplicates for each condition, and only NO_3_-N and DRP were measured. For this, water was supplied to the bottom of two parallel columns at a flow rate of 6 mL/min, which was maintained by adjusting the inflow water height to achieve the target flow by creating a hydraulic head of approximately 3 cm between a 20-L tank (with DI or SSW) and the experimental columns, with no differences between them (Fig. 1). Each test was run for 90 min, with an initial 30-min stabilization period, after which samples were taken at 15-min intervals (i.e., samples taken at 30, 45, 60, 75, and 90 min). Samples were collected from a sampling point in each water column 2 cm above the sediment (Fig. 1). The experiments were run three times on different dates.

### Flume design and experimental methodology

**Fig. 2:**
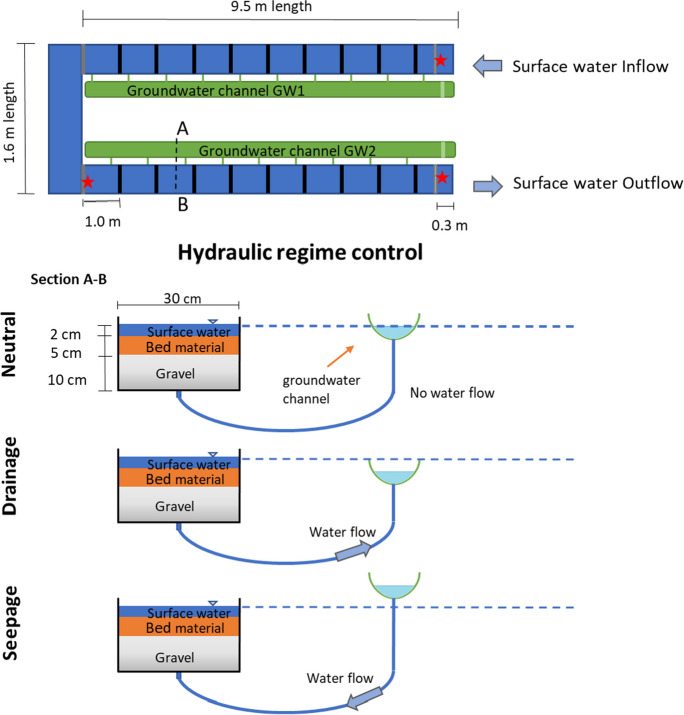
Schematic layout and cross-sectional representations of the experimental setup, illustrating the controls of the groundwater hydraulic regime. The stars indicate the locations for sampling and in situ monitoring

A 19-m long flume, divided into two main sections each of 9.5 m, was constructed using 6-mm thick polyvinyl chloride (PVC) sheets. This setup simulated an in-channel treatment system containing gravel, bed sediment, surface water, and a mechanism for managing groundwater interactions (Fig. 2). The initial section of the flume was connected to the groundwater channel GW1 with 10 tubes spaced 1 m apart for the hydraulic regime control, while the second section contained 9 tubes connected to the groundwater channel GW2 (see Figure A2 in Supplementary Information). The flume’s length was determined by the maximum available laboratory space to promote enough time and area for sediment–water interactions. A 0.5% slope was set to represent a typical hydraulic gradient in a system with minimal backflow. The hydraulic regime (i.e., groundwater seepage, neutral or drainage water conditions) was varied by adjusting the groundwater channel (GW1 and GW2) height relative to the flume height via a pulley system. The surface water and groundwater used in this experiment were the same synthetic stormwater (SSW) previously described.

Grade 5 gravel (5–8 mm diameter) was used to represent free-draining gravel in the lower hyporheic zone, while the bed media consisted of mixtures of sand and contaminated bed sediment as previously described. A layer of cheesecloth (cotton, 28 by 24 threads per square inch) was used between the layers to prevent the migration of fine particles into the gravel.

The flume was gravity-fed to achieve a low water level (1 cm depth) with an inflow rate of 7.2 L/min of SSW and a high-water level (2 cm depth) with an inflow of 21.8 L/min (water velocity of 0.04 and 0.06 m/s, respectively). A flow sensor (electromagnetic flow meter Krohne Optiflux 4100) was installed at the outlet of the stormwater feeder tank intermediate bulk containers (IBC) that were used to monitor the target flows.

No interaction between surface water and groundwater occurred under neutral groundwater conditions. To induce drainage conditions, the groundwater channel system was lowered to facilitate draining 10% of the surface water flow (1.42 L/min under 1 cm water depth and 4.36 L/min under 2 cm water depth of the incoming surface water) for each length of the flume (GW1 and GW2 as per Fig. 2). Under groundwater seepage conditions, the groundwater channel was raised to seep SSW into the flume at a total rate of 20% of the surface water flow. The composition of inflowing groundwater under seepage conditions was the same as the surface water to avoid dilution. The groundwater flow was measured by collecting a known volume of water in a volumetric cylinder while recording the time taken to fill using a chronometer. The flow rate remained nearly constant throughout the measurement, with minor adjustments made during the experiment to maintain steady flow conditions.

### Flume simulations

Experiments were run under three different groundwater conditions (neutral, drainage water, and groundwater seepage), two different surface water depths (1 and 2 cm) and corresponding flows (7.2 L/ min and 21.8 L/ min, respectively), and with three-bed sediment compositions (only gravel, 40% sediment + 60% sand—high hydraulic conductivity, and 75% sediment + 25% sand—low hydraulic conductivity; Table 1). The proportion of 40% sediment and 60% sand was determined based on the results of the hydraulic conductivity test (Appendix Tables A3 and A4). This composition was chosen for its ability to increase the proportion of WRB’s bed sediment within the range of 1:4 to 1:1 (WRB sediment to sand), thus achieving the desired hydraulic conductivity for the flume. Additionally, this mixture incorporates a significant portion of WRB bed sediment while assuring the ability to seep/drain 20% of surface water flow. The residence time of water in the sediment was calculated by interpolation and extrapolation of the results obtained using the column leaching experiment.

**Table 1:** Groundwater condition, water depth and flow, and type of bed sediment used in flume experiments

Groundwater condition	Water depth and surface water flow (L/min)	Bed sediment	Number of runs
Neutral	Low (1 cm and 7.2 L/min)	Gravel only	1
	High (2 cm and 21.8 L/min)		1
Drainage	Low		1
	High		1
Seepage	Low		1
	High		1
Neutral	Low	High hydraulic conductivity: 40% WRB sediment and 60% silica sand w/ gravel layer	3
	High		3
Drainage	Low		3
	High		3
Seepage	Low		3
	High		3
Neutral	High	Low hydraulic conductivity: 75% WRB sediment and 25% silica sand with gravel layer	3
Drainage	High		3
Seepage	High		3
Neutral	High	New mix of low hydraulic conductivity: 75% WRB sediment and 25% silica sand with gravel layer	2
Drainage	High		2
Seepage	High		2
Total of runs performed			39

Between runs, the gravel layer and bed sediment remained saturated with SSW. However, prior to each run, a fresh SSW was applied to replace the SSW from the previous run, and the system was allowed to stabilize for 15 min prior to sample collection (Table 1). Experiments with low hydraulic conductivity sediment and high surface water flow were repeated twice due to high variance in nutrient concentrations during the study and potential accumulation of nutrients in the bed sediment throughout the experiment. The flume’s bed sediment was only removed and replaced for four major experiments as indicated in Table 1.

### Sample collection

Samples were taken from the inlet, midpoint, and outlet of the flume (Fig. 1) using 1-L high-density polyethylene (HDPE) containers and stored at 4 °C before analysis. Inlet water quality was sampled at 10-min intervals from the start of the flume trial (*t* = 0). Middle and outlet concentrations were found to stabilize after 15 min. Therefore, samples were taken from the midpoint and outlet of the flume at 10-min intervals, starting at *t* = 15 min. Groundwater samples were collected at *t* = 15 and 35 in the first channel and *t* = 25 and 45 min in the second channel under groundwater seepage. Under drainage conditions, groundwater samples were collected at *t* = 15, 25, 35, and 45 min from both groundwater channels. More samples were taken under drainage conditions as a greater change in groundwater quality was expected than under groundwater seepage, where the groundwater was expected to change from its synthetic stormwater source. Samples were filtered with a 0.45-µm filter and frozen at − 18 °C for storage before processing.

### Method of analyses

NO_3_-N and DRP were then analyzed with a Dionex ICS-2100 ion chromatography (IC) system using 38 mM KOH as eluent at 0.3 mL/min, with the preserved samples thawed at room temperature directly prior to IC analysis. Ammoniacal nitrogen was analyzed using flow injection analysis with a modified method based on Foss A/N 5206 & 5232. The detection limit for this method was 0.10 mg/L.

For the in situ monitoring in the flume, a YSI Professional Plus multi-parameter meter was positioned at each collection point to record pH, electrical conductivity, oxidation–reduction potential (ORP), and temperature data at 1-min intervals during each run. ORP values were converted to standard hydrogen electrode (SHE), displaying redox potential as Eh (V) by adding 200 mV to the ORP values (Environmental, 2005).

### Quality control and quality assurance

A QA/QC plan was implemented to minimize errors in data analysis. All samples were collected, preserved, and analyzed following the APHA (2007) guidelines. Duplicates were generated for ammoniacal nitrogen, oxidized nitrogen, and DRP. Sampling instruments were calibrated and maintained according to the manufacturer’s manual.

### Statistical analysis

To account for variation in inlet concentration, samples’ nutrient concentrations taken in the midpoint and outlet of the flume were evaluated against the mean inlet levels for each run. The resulting percent changes in each contaminant concentration were then evaluated across all runs. *T*-tests (with α = 0.05) were performed to assess statistically significant differences in the percentage changes of each dataset under varying groundwater conditions, aiming to determine their effect on the surface water quality.

## Results

### Change in nitrate and DRP over time under column leaching conditions

Water resident time in the sediment in the column ranged from 90 s using 4 cm of sediment mix and 1 cm water depth to 217 s with 5 cm of sediment mix and 2 cm of water depth using 50% WRB sediment and 50% sand and between 21 and 40 s using 20% WRB sediment and 80% silica sand (Table A5).

**Fig. 3:**
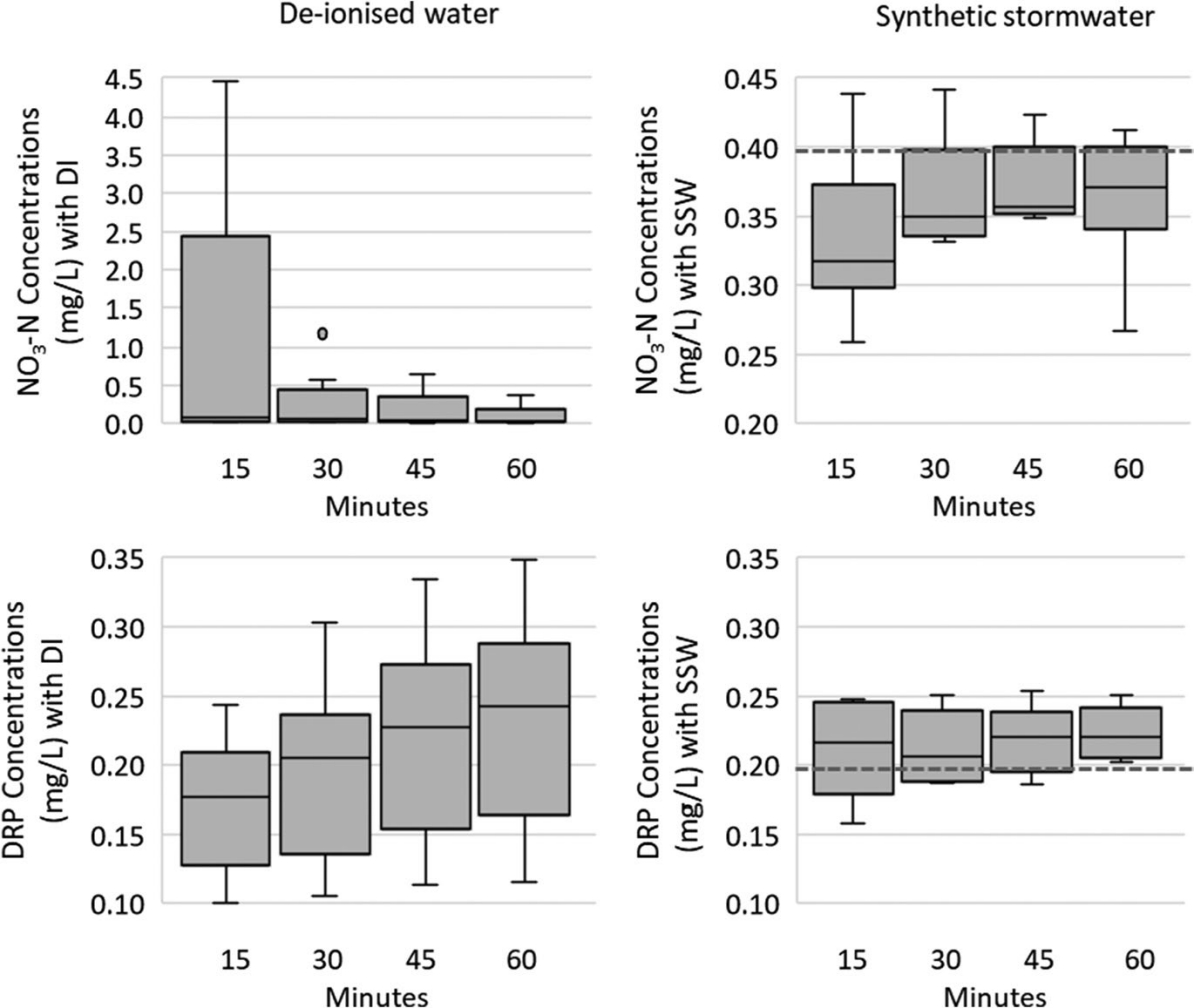
Water collected on the column leaching with sediment mix ratio 50% WRB and 50% sand test at times 15, 30, 45, and 60 min, respectively. Dotted line represents SSW concentrations of 0.4 mg/L NO_3_-N and 0.2 mg/L DRP

Under DI water application with a sediment mix ratio of 50% WRB and 50% sand, there was an initial high export of NO_3_-N with concentrations up to 4.4 mg/L which then decreased to less than 0.5 mg/L after 60 min (Fig. 3, Table A6). Subsequent tests resulted in mean concentrations close to or below the detection limit (0.045 mg/L). Under SSW application, nitrate concentrations at the outlet were initially below the SSW concentration of 0.4 mg/L NO_3_-N and later increased to similar values to SSW concentrations. For DRP, however, DI water application through the media showed mean DRP concentrations increasing over time from 0.17 to 0.24 mg/L, achieving the highest concentration of 0.348 mg/L. Under SSW application, leachate concentrations were slightly higher than the influent concentrations (Fig. 3; Table A6).

### Comparison of hydraulic conductivity characteristics of bed sediment on nutrient dynamics

**Fig. 4:**
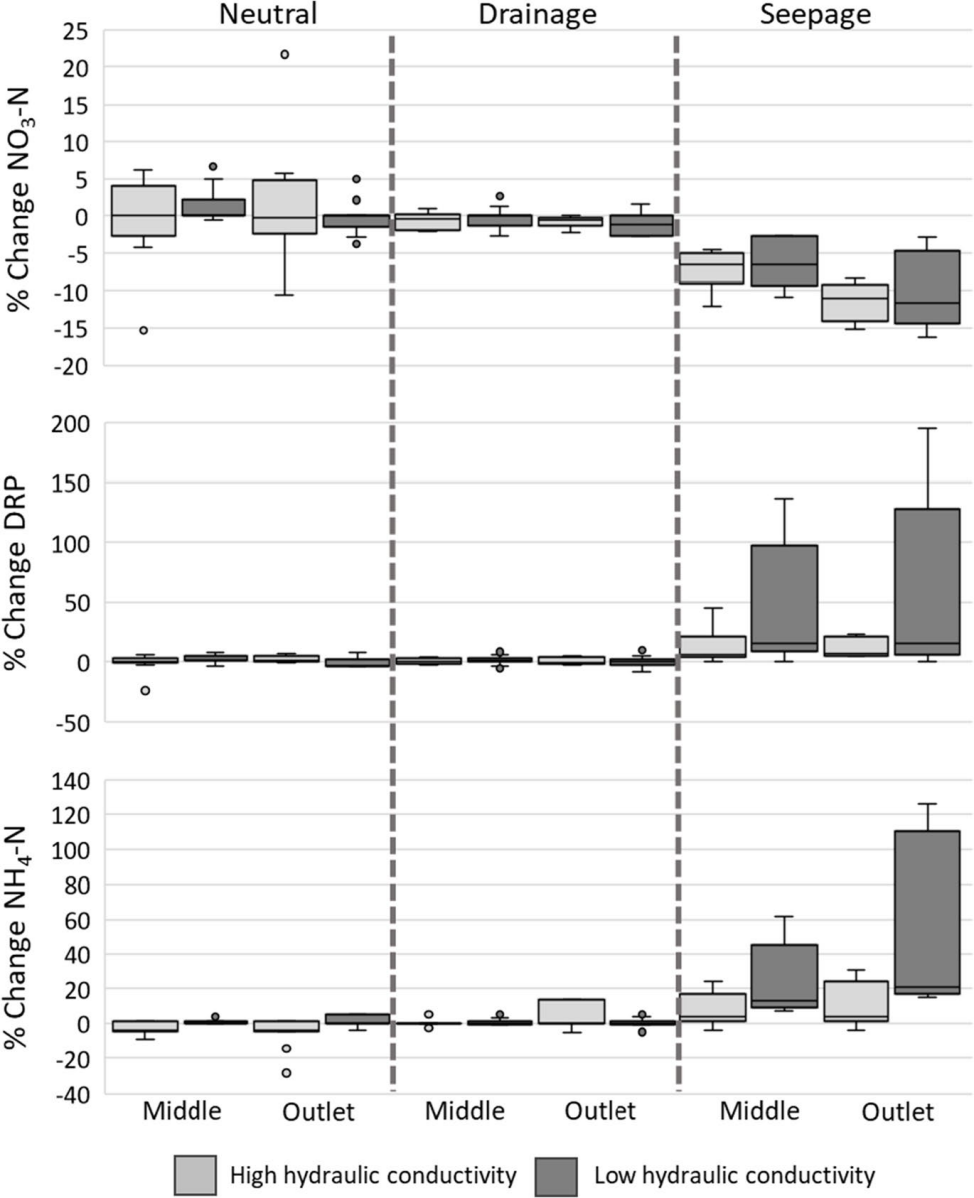
Percentage changes in NO_3_-N, DRP, and NH_4_-N concentrations between inlet, at the midpoint and outlet of the flume under neutral, drainage, and groundwater seepage conditions; outliers are indicated by circles

Residence time of each high hydraulic conductivity was estimated as 67 s (for low water level) and 119 s (for high water level). Residence time for low hydraulic conductivity sediment was estimated as 218 s and 147 s, for low and high water levels, respectively (see Table A5, Supplementary Information). Both low and high hydraulic conductivity sediments were found to have a similar type of influence on nitrogen and phosphorus, i.e., both resulted in the reduction of NO_3_-N and increase of NH_4_-N and DRP concentrations in surface water under groundwater seepage conditions. However, the scale of the change and range of variation in concentrations was much larger for the low-conductivity sediment (Fig. 4, Table 2).

**Table 2:** Results of NO_3_-N, NH_4_-N, and DRP concentrations in the inlet, midpoint, and outlet of the flume using low and high hydraulic conductivity sediment under different groundwater conditions

Sediment hydraulic conductivity	GW condition	Location	Observations	NO_3_-N Average and range (mg/L)	NH_4_-N Average and range (mg/L)	DRP Average and range (mg/L)
High	Neutral	Inlet	10	0.39 (0.37–0.41)	0.19 (0.17–0.21)	0.20 (0.18–0.22)
		Middle	11	0.39 (0.36–0.43)	0.18 (0.17–0.20)	0.20 (0.19–0.22)
		Outlet	11	0.38 (0.36–0.41)	0.18 (0.15 –0.20)	0.20 (0.20–0.22)
	Drainage	Inlet	12	0.38 (0.36–0.40)	0.16 (0.12 –0.18)	0.21 (0.20–0.22)
		Middle	12	0.38 (0.37–0.39)	0.16 (0.12–0.18)	0.21 (0.20–0.22)
		Outlet	12	0.38 (0.39–0.39)	0.17 (0.14–0.18)	0.21 (0.19–0.22)
		Groundwater	22	0.14 (0.08–0.18)*	0.18 (0.11–0.30)*	0.43 (0.04–0.68)*
	Seepage	Inlet	12	0.37 (0.36–0.39)	0.17 (0.13–0.19)	0.20 (0.19–0.20)
		Middle	12	0.34 (0.32–0.37)*	0.18 (0.16–0.19)*	0.22 (0.20–0.29)*
		Outlet	12	0.33 (0.31–0.36)*	0.18 (0.17–0.19)*	0.22 (0.20–0.25)*
		Groundwater	12	0.36 (0.34–0.39)	0.19 (0.18–0.20)	0.17 (0.12–0.19)
Low^1^	Neutral	Inlet	20	0.39 (0.35–0.43)	0.25 (0.24–0.25)	0.20 (0.19–0.21)
		Middle	22	0.40 (0.36–0.44)	0.25 (0.25–0.26	0.21 (0.19–0.22)
		Outlet	22	0.40 (0.35–0.43)	0.25 (0.24–0.26	0.21 (0.19–0.22)
	Drainage	Inlet	20	0.39 (0.36–0.42)	0.25 (0.24–0.26)	0.21 (0.20–0.22)
		Middle	25	0.38 (0.36–0.42)	0.25(0.25–0.26)	0.21 (0.20–0.23)
		Outlet	25	0.38 (0.36–0.42)	0.25(0.24–0.26)	0.21 (0.20–0.23)
		Groundwater	40^2^	0.58 (0–3.36)*	1.02 (0.66–1.50)*	2.28 (0.18–6.16)*
	Seepage	Inlet	20	0.38 (0.37–0.40)	0.25 (0.24–0.26)	0.21 (0.18–0.24)
		Middle	25	0.35 (0.33–0.37)*	0.31 (0.27–0.40)*	0.31 (0.20–0.45)*
		Outlet	25	0.34 (0.31–0.35)*	0.38 (0.29–0.56)*	0.34 (0.22–0.56)*
		Groundwater	20^2^	0.37 (0.36–0.40)	0.25 (0.24–0.25)	0.22 (0.18–0.28)

*Significant statistical difference between inlet concentrations (*P*-value < 0.05) ^1^NH_4_-N had 12 observations at all inlet sampling and under all neutral conditions and 15 observations at the middle and outlet under drainage and seepage conditions ^2^NH_4_-N observations were 24 and 12, respectively

In contrast, the high-conductivity sediment produced greater variation in Eh and pH under groundwater seepage conditions (Fig. 6). The decrease in Eh values observed under groundwater seepage conditions indicates potential anoxic bed sediment conditions.

**Fig. 5:**
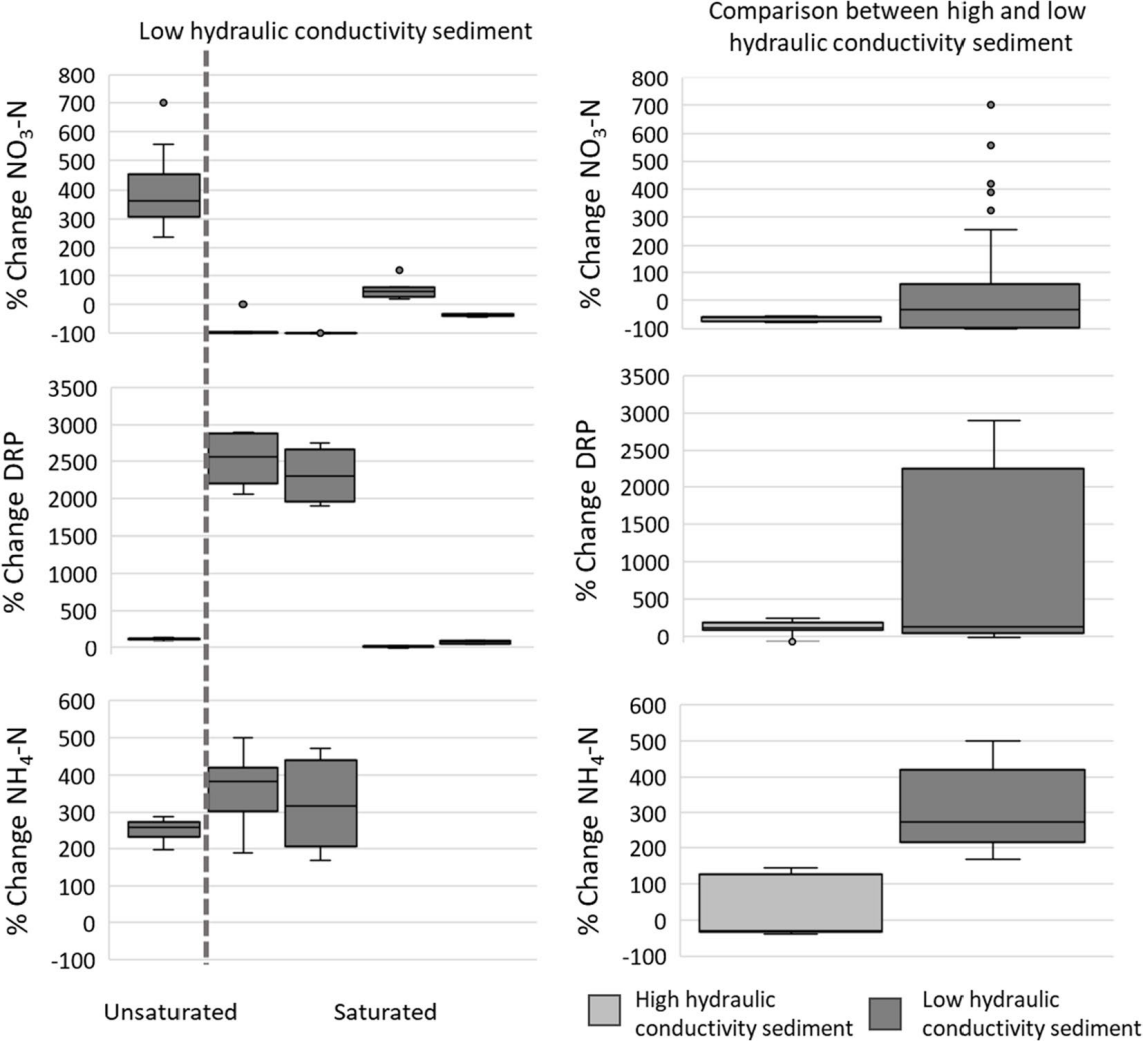
Percentage change in NO_3_-N, DRP, and NH_4_-N concentrations between inlet and in the groundwater channel under drainage water condition. NH_4_-N concentrations were not analyzed in the last two runs; outliers are indicated by circles

Water collected at the groundwater channel under drainage water conditions had an increase of NO_3_-N on the first run; however, there was almost a 100% reduction of NO_3_-N on the following runs (Fig. 5). DRP concentrations, on the contrary, showed a greater increase on runs 2 and 3 compared to the first run, with percentage changes going from around 100% increase to up to 2500% increase (Fig. 5).

### Pollutant dynamics along a flume channel under varied groundwater conditions

Results for high water level conditions in the flume showed that the GW condition influenced nutrient levels in the surface water for NO_3_-N, NH_4_-N, and DRP (Table 2, Fig. 4). Under groundwater seepage conditions, NO_3_-N levels were reduced by 8% at the midpoint and 11% at the outlet, whereas NH_4_-N and DRP concentrations rose by 10 and 11%, respectively, at both locations, relative to the inlet concentrations, when using high hydraulic conductivity sediment. Using low hydraulic conductivity sediment, NO_3_-N levels were reduced by 11% at the outlet, and NH_4_-N and DRP concentrations were raised by 51% and 65%, respectively. Under neutral and drainage conditions, no significant difference in percentage change was observed (Table 2). Results of pollutant dynamics under low water levels were inconsistent and nutrient concentrations under different groundwater conditions did not exhibit any significant trend.

Under drainage water conditions, substantial shifts were noticed in sample concentrations taken from the groundwater channel, with a sharp decline in NO_3_-N levels and an increase in DRP concentrations. The increase was greater using low hydraulic conductivity sediment (Fig. 7, Table 2). The decrease in NO_3_-N suggests that it is retained within the bed sediment as surface water flows through it under drainage water conditions, whereas DRP appears to be released or flushed out from the bed sediment. In all runs, there was an export of NH_4_-N. It is important to note that the bed was water-saturated (using SSW) during all runs except during the first run, where the bed sediment/gravel was unsaturated because the flume was previously drained and not fully filled before this initial run (Fig. 5).

### pH, conductivity, Eh, and temperature changes throughout channel over time

**Fig. 6:**
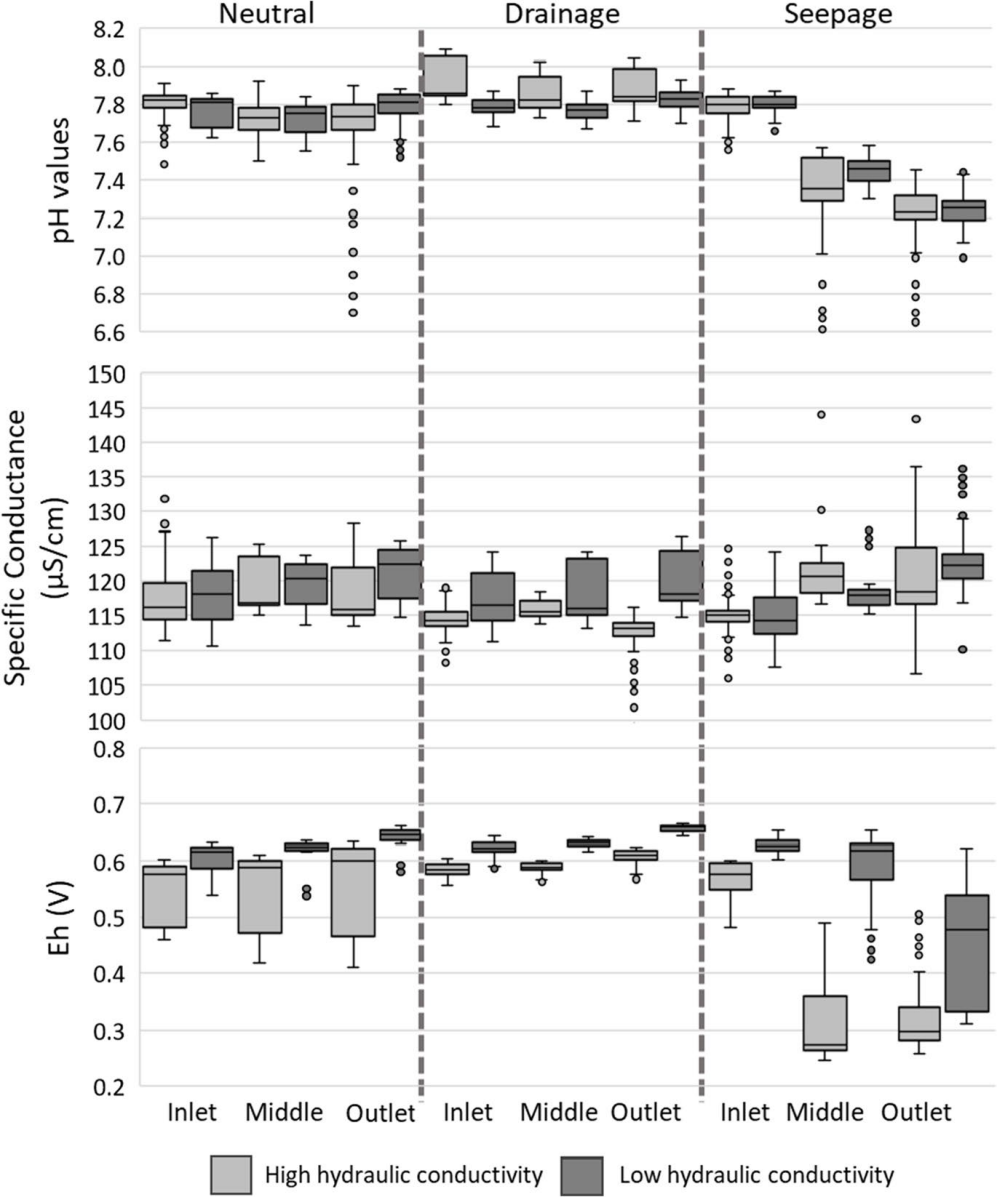
pH, specific conductivity, and Eh from the in situ measurement after the stabilization period; outliers are indicated by circles

pH, specific conductance, and Eh changed under groundwater seepage conditions. The average inlet pH values decreased from 7.8 to 7.4 at the midpoint and 7.3 at the outlet (Fig. 6). Specific conductivity remained consistent, ranging between 106 and 132 µS/cm, with average values between 112 and 121 µS/cm.

Under seepage conditions, the mean Eh values at the inlet, middle, and outlet were around 0.6 V, 0.5 V, and 0.3 V, respectively, using low and high hydraulicity conductivity sediment. The 0.3 V reduction in average Eh values under groundwater seepage conditions, with just a 20% input from groundwater, suggests anoxic conditions within the bed sediment. Temperature showed no specific pattern, with a mean of 17.5 °C and a range between 16 and 19 °C.

## Discussion

### Implications for nutrient dynamics in waterways and in-channel treatment systems

Saturating the flume’s bed sediment with SSW likely established an anoxic environment, supporting denitrification processes to occur, as suggested by the Eh values observed under groundwater seepage conditions. Each flume run, lasting up to 120 min (including preparatory steps such as replacing the water in the water-saturated bed sediment and applying a 30-min flow through the flume), might have allowed anoxic zones to persist within the bed sediment, facilitating denitrification processes. However, given the short resident time of the water within the sediment (ranging from 67 to 218 s; Table A5), denitrification alone cannot account for the observed reduction in NO_3_-N concentrations by 11% under groundwater seepage conditions (Fig. 6, Table 2). Sandy bed sediments have non-uniform (preferential) groundwater flow, which might have prevented all water in the bed sediment from being replaced before the start of each run on the flume experiment (MahmoodPoor Dehkordy et al., 2019). Thus, denitrification, mixing, and dilution of SSW seeping from the bed sediment within the flume could account for the 11% reduction in NO_3_-N levels in the surface water under groundwater seepage conditions.

The ammonification processes of organic matter releases ammonia, which can later be transported from the pore water in the sediment bed to the surface water. The observed release of ammonia under seepage conditions to the surface water and under drainage conditions to the groundwater channel contributes to this understanding (Fig. 6, Table 2). Nitrite and nitrogen oxide are also generated within small streams and channels, but this was not measured nor quantified in this research.

Groundwater seepage induced the physical transport of NH_4_-N and DRP from the bed-sediment porewater into the surface water due to the upward flux of water, increasing the concentrations of NH_4_-N and DRP in the surface water by between 10–51% and 11–65%, respectively (Fig. 7, Table 2). Conversely, the release of NH_4_-N and DRP into groundwater under drainage conditions aligns with prior findings where phosphorus leaching under groundwater seepage conditions increased phosphorus load into the surface water (Yoder, 2014). Consequently, the water draining through the bed sediment facilitated the transport of ammoniacal nitrogen and phosphorus, subsequently leaching from the bed sediment into the groundwater channel.

### Influence of sediment conditions on nitrogen and phosphorus transformation

Low hydraulic conductivity sediment induced greater changes in nutrient concentration, probably due to the higher proportion of WRB content, which contained more organic matter and nutrients in the sediment mix, and, under ammonification and leaching processes, could export more NH_4_-N and DRP to both surface water (average increase of 52% and 65%, respectively) and groundwater (average increase of 300% and 1000%, respectively) (Figs. 6 and 7, Table 2). High hydraulic conductivity sediment led to more pronounced variations in Eh and pH due to the greater content of sand, which facilitated the mixing of pore water with both surface water and groundwater.

Changes in Eh values (from 0.6 to around 0.25 V, Fig. 6) observed using both low and high hydraulic conductivity sediment suggest that conditions go from aerobic to anoxic (or perhaps anaerobic in micro-sites) in the bed sediment, which could have influenced microbial activity when the flume had water-saturated bed sediment. This supports denitrification processes, highlighting the importance of saturated bed sediment in the nitrogen cycle.

Water-saturated sediment was an important parameter to reduce nitrate concentrations in the surface water (Fig. 7, Table 2). The first run of bed sediment was not saturated with SSW (unsaturated aerobic conditions), but the following runs had the bed sediments saturated, suggesting water saturation of bed sediment can change the pollutant’s dynamics (Silveira et al., 2021). Water-saturated sediment is the main difference between dry vs. wet basin, where dry basin might not promote denitrification processes as well as wet basins.

The elevated phosphorus content in the bed sediment is likely due to the catchment’s historical phosphorus inputs (Moores et al., 2009), which can subsequently be released into surface water. Therefore, characterizing bed sediment is essential for the development of in-channel treatment and effective management planning.

### Impact of findings

Groundwater seepage conditions contribute significantly to the dynamics of nutrients in surface water, and thus, it is important to consider both natural and anthropogenic groundwater variations when assessing surface water quality. Locations where groundwater can experience reductions during extended periods without rain, seasonal droughts, and excess of groundwater extraction should be identified as they can change groundwater levels and increase nutrient concentrations. Conversely, prolonged periods of rain and sea-level rise can elevate groundwater levels in low-lying catchments and unconfined coastal areas, impacting in-channel treatment systems within these regions.

Additionally, the assessment of hydraulic conductivity in bed sediment is important, given the significant difference in nutrient dynamics as observed when using low vs. high hydraulic conductivity bed sediment. Furthermore, the saturation of bed sediment in in-channel treatment systems emerges as a significant factor influencing nutrient dynamics, particularly in regions where elevated nitrate in surface groundwater is a concern. For example, sea-water level rise could affect groundwater seepage within low-lying streams, which could increase loads of nitrate in the surface water. All these factors should be taken into account in the decision-making process when designing in-channel treatment systems that involve interactions between groundwater and surface water.

In-channel treatment systems, (i.e., engineered systems) can be designed with a range of bed materials, control surfaces, and drainage systems to regulate hydraulic properties to optimize treatment processes. In addition, knowledge of the pre-modified groundwater regime of a stream can help determine the frequency of contaminated bed material removal and treatment efficiency of unmodified streams. Adaptive management practices, driven by real-time monitoring and informed by research, can significantly improve the long-term effectiveness and maintenance schedule for these systems. Ultimately, combining insights from natural channels with engineered solutions can lead to a more resilient and efficient in-channel treatment system.

### Limitations

This laboratory-based study aimed to isolate processes occurring among surface water, groundwater, and bed sediment to evaluate the effects of various groundwater conditions (seepage, neutral, and drainage) and varying bed sediment hydraulic conductivities on nitrogen and phosphorus dynamics within in-channel treatment systems. Inconsistent results under low water levels can likely be attributed to difficulties around controlling a low groundwater flow rate of 0.72 L/min in or out the flume. The exchange of new water after the previous test may have been slower and more variable in time required under low flow conditions with less driving head, resulting in only partial exchange within the 15-min stabilization period.

It was assumed that the bed sediment within the flume was homogeneous and that consistent groundwater conditions prevailed throughout the experimental setup. The flat streambed topography used in the flume experiment would limit the hyporheic exchange at the sediment–water interface, as the increase of bedforms’ height difference influences the hyporheic exchange pattern (Jin et al., 2022b). Further research on the influence of non-submerged streambed structures, including variable bedforms such as riffles is needed to enhance our understanding of surface-to-groundwater interactions in a wider range of in-channel treatment systems.

The study did not account for other biotic and abiotic processes involving organisms such as fauna, flora, and photosynthesis, as well as physical processes such as sunlight and rainfall. Additionally, the use of synthetic stormwater, achieved by adding nutrient salts, may not fully capture the complexity of natural stormwater, which encompasses diverse substances, temperature variations, and fluctuating flow rates.

## Conclusions

This study highlights the significant role of groundwater interaction on nutrient dynamics in surface water, with direct implications for engineering interventions for the sustainable management of groundwater and surface water systems. There was an observed pattern under seepage conditions, where NO_3_-N concentrations in the surface water decreased due to denitrification, mixing, and dilution; NH_4_-N concentrations increased due to ammonification and leaching from bed sediment; and DRP concentrations increased due to leaching from bed sediment, alongside shifts in pH and Eh.

Furthermore, low-conductivity sediment induces greater changes in nutrient concentration, while high-conductivity sediment leads to more pronounced variations in Eh and pH. Additionally, the level of bed sediment saturation with stormwater influences nutrient dynamics and thus highlights the importance of incorporating this parameter into engineering strategies for improved nutrient prediction and removal.

The lessons learned from this research can be applied to a broad range of engineered in-channel systems where groundwater and surface water interact, and nutrient contaminants are of concern. This is particularly relevant where sea-water levels could affect groundwater levels in low-lying streams.

## Supplementary Information

Below is the link to the electronic supplementary material.

ESM 1 (DOCX 600 KB)

## Data Availability

The data supporting the findings of this study are available within the paper and its Supplementary Information files. Additional data, if needed, are available from the corresponding author upon reasonable request.
