# Supplementary material for: The influence of surface–groundwater interactions on nutrient dynamics in urban in-channel treatment systems
Source: Environ Monit Assess. 2024 Dec 11;197(1):51. doi: 10.1007/s10661-024-13459-4 (PMC11634930; doi:10.1007/s10661-024-13459-4)
Supplement: Supplementary file 1 — (DOCX 600 KB) [file 10661_2024_13459_MOESM1_ESM.docx]

Supplementary Information


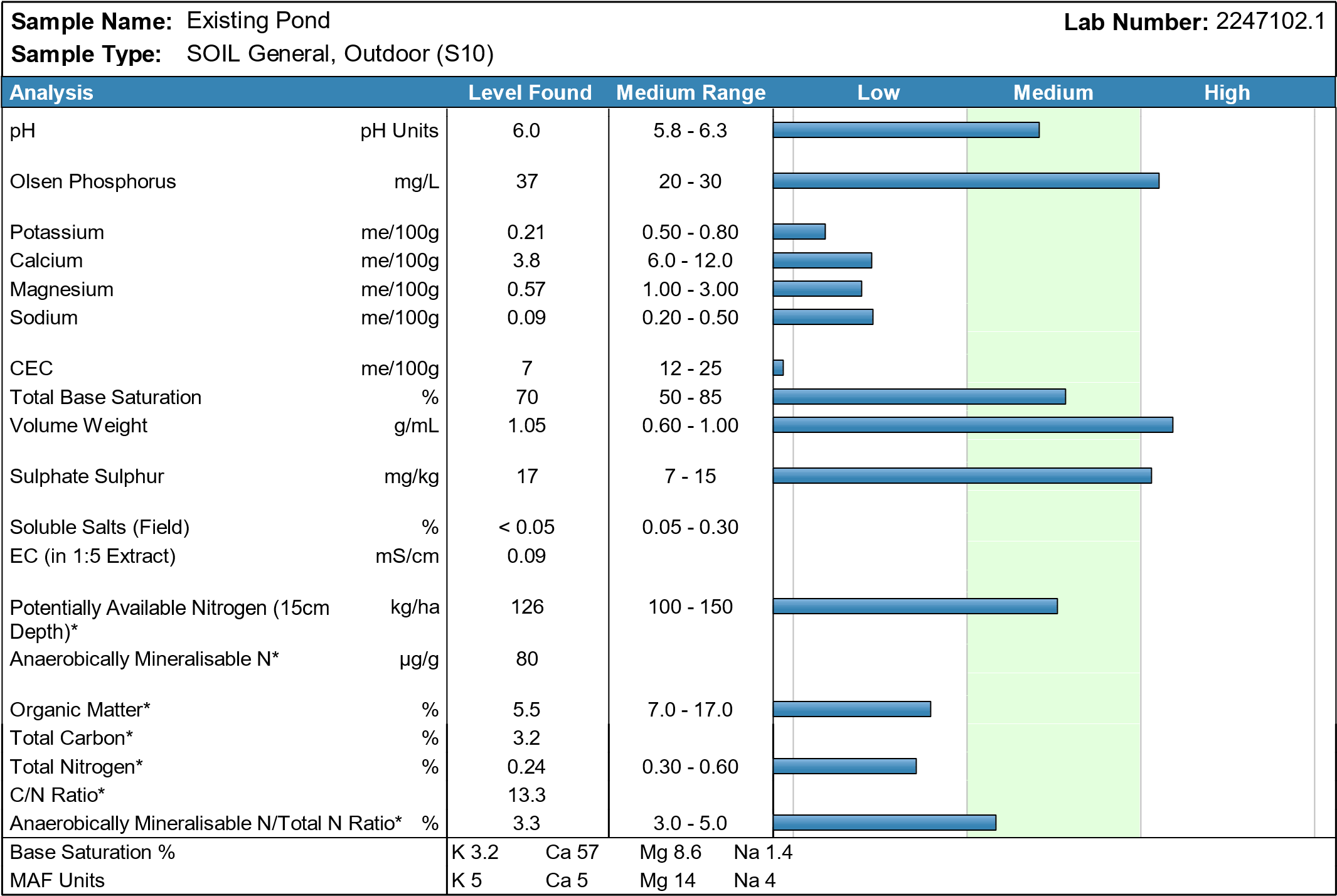


Figure A1. Wigram Basin summary of soil analysis


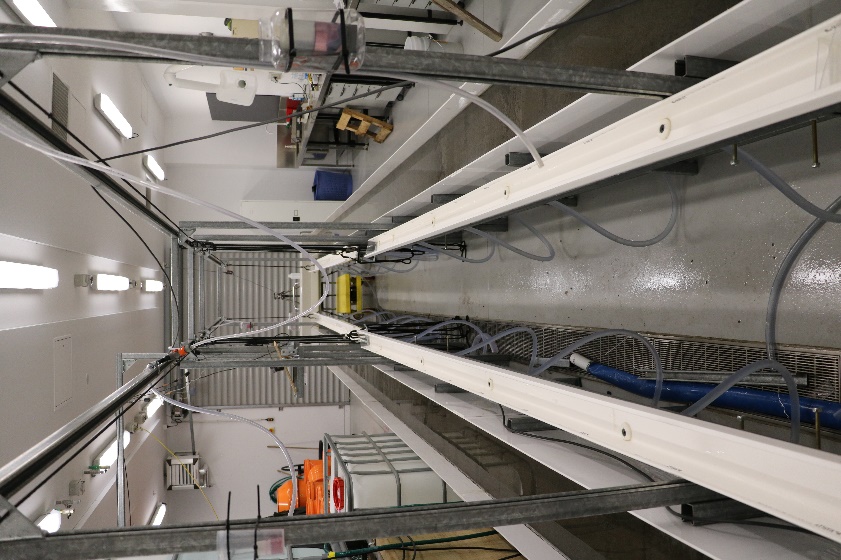

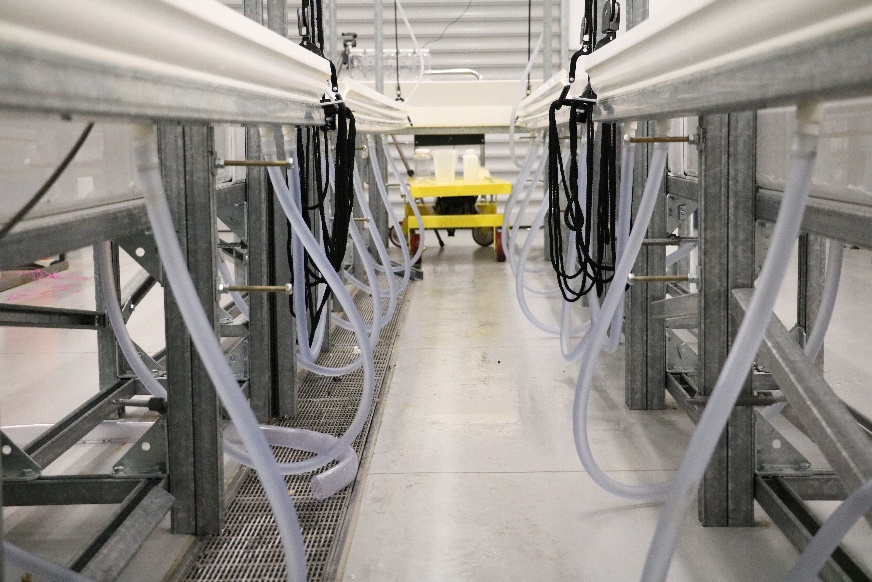


Figure A2: Picture of controls of the groundwater hydraulic regime. Left picture shows the flume from the top where both surface water and groundwater channels can be seen. Right picture shows the bottom where a total of 19 pipes connected both surface water and groundwater channels.

| **Soil Analysis Results** | | |  |
| --- | --- | --- | --- |
| pH | pH Units | | 6.0 |
| Olsen Phosphorus | mg/L | | 37 |
| Potassium | me/100g | | 0.21 |
| Potassium | %BS | | 3.2 |
| Potassium | MAF units | | 5 |
| Calcium | me/100g | | 3.8 |
| Calcium | %BS | | 57 |
| Calcium | MAF units | | 5 |
| Magnesium | me/100g | | 0.57 |
| Magnesium | %BS | | 8.6 |
| Magnesium | MAF units | | 14 |
| Sodium | me/100g | | 0.09 |
| Sodium | %BS | | 1.4 |
| Sodium | MAF units | | 4 |
| CEC | me/100g | | 7 |
| Total Base Saturation | % | | 70 |
| Volume Weight | g/mL | | 1.05 |
| Sulphate Sulphur | mg/kg | | 17 |
| Soluble Salts (Field) | % | | < 0.05 |
| EC (in 1:5 Extract) | mS/cm | | 0.09 |
| Potentially Available Nitrogen kg/ha (15cm Depth) | | | 126 |
| Anaerobically Mineralisable N* µg/g | | | 80 |
| Organic Matter* | | % | 5.5 |
| Total Carbon* | | % | 3.2 |
| Total Nitrogen* | | % | 0.24 |
| C/N Ratio* | |  | 13.3 |
| Anaerobically Mineralisable N/Total% N Ratio* | | | 3.3 |

Table A1. Detailed soil chemical analysis results for Wigram Basin

| Summary of Methods | |  |
| --- | --- | --- |
| **Test** | **Method Description** | **Default Detection Limit** |
| Soil Prep (Dry & Grind)* | Air dried at 35 - 40°C overnight (residual moisture typically 4%) and crushed to pass through a 2mm screen. | - |
| pH | 1:2 (v/v) soil:water slurry followed by potentiometric determination of pH. In-house. | 0.1 pH Units |
| Olsen Phosphorus | Olsen extraction followed by Molybdenum Blue colorimetry. Inhouse method. | 1 mg/L |
| Sulphate Sulphur | 0.02M Potassium phosphate extraction followed by Ion Chromatography. In-house. | 1 mg/kg |
| Potentially Available Nitrogen* | Determined by NIR, calibration based on Available N by Anaerobic incubation followed by extraction using 2M KCl followed by Berthelot colorimetry. (Calculation based on 15cm depth sample). Note that any Mineral N present is included in the AN/AMN result reported. | 1 mg/L |
| Anaerobically Mineralisable N* | As for Potentially Available Nitrogen but reported as µg/g. | 5 µg/g |
| Organic Matter* | Organic Matter is 1.72 x Total Carbon. | 0.2 % |
| Soluble Salts (Field) | 1:5 soil:water extraction followed by potentiometric determination of conductivity. Calculated by EC (mS/cm) x 0.35. In-house. | 0.05 % |
| Electrical Conductivity (EC) | Electrical Conductivity measured in 1:5 Soil:Water extract. | 0.01 mS/cm |
| Total Carbon* | Determined by NIR, calibration based on Total Carbon by Dumas combustion. | 0.1 % |
| Total Nitrogen* | Determined by NIR, calibration based on Total N by Dumas combustion. | 0.04 % |
| Potassium | 1M Neutral ammonium acetate extraction followed by ICP-OES. In-house. | 0.01 me/100g |
| Calcium | 1M Neutral ammonium acetate extraction followed by ICP-OES. In-house. | 0.5 me/100g |
| Magnesium | 1M Neutral ammonium acetate extraction followed by ICP-OES. In-house. | 0.04 me/100g |
| Sodium | 1M Neutral ammonium acetate extraction followed by ICP-OES. In-house. | 0.05 me/100g |
| CEC | Summation of extractable cations (K, Ca, Mg, Na) and extractable acidity. May be overestimated if soil contains high levels of soluble salts or carbonates. In-house. | 2 me/100g |
| Total Base Saturation | Calculated from Extractable Cations and Cation Exchange Capacity. | 5 % |
| Volume Weight | The weight/volume ratio of dried, ground soil. In-house. | 0.01 g/mL |

Table A2. Soil analysis methods.

| **Compound** | F-  (ppm) | Cl-  (ppm) | SO_4_^2-^ (ppm) | NO_3_-N  (ppm) | NH_4_-N  (ppm) | DRP  (ppm) | Na (ppb) | Mg  (ppb) | Al  (ppb) | K  (ppb) | Ca  (ppb) | Cr (ppb) | Fe (ppb) | Mn (ppb) | Ni (ppb) | Cu (ppb) | Zn (ppb) | Pb (ppb) |
| --- | --- | --- | --- | --- | --- | --- | --- | --- | --- | --- | --- | --- | --- | --- | --- | --- | --- | --- |
| **Average concentration** | <1.0 | 5.1 | 4.4 | 0.39 | 0.18 | 0.20 | 5373.7 | 1455.1 | 103.9 | 1062.6 | 11909.0 | 9.3 | 5.7 | 0.4 | 36.6 | 2.4 | 205.5 | 0.7 |
| **Maximum concentration** | <1.0 | 5.9 | 8.5 | 0.36 | 0.26 | 0.22 | 7234.5 | 1880.3 | 4741.7 | 1336.3 | 23276.7 | 226.5 | 184.7 | 4.7 | 963.4 | 3.8 | 257.9 | 19.8 |
| **Minimum concentration** | <1.0 | 1.8 | 1.3 | 0.42 | 0.13 | 0.18 | 4134.1 | 1106.0 | 21.5 | 897.4 | 8214.4 | 0.5 | 0.0 | 0.0 | 0.1 | 1.8 | 157.4 | 0.1 |

Table A3: Composition analysis of SSW inlet concentration.

Table A4: Results of hydraulic conductivity test between soil mix and soil depth. Red shows very relevant and green shows relative statistically difference.

| **Criteria** | **Condition** | **Number of samples** | **Mean hydraulic conductivity (cm/s)** | **Variance** | **P-value** |
| --- | --- | --- | --- | --- | --- |
| Soil mix | 50% WRB sediment / 50% sand | 18 | 3.2E-02 | 2.0E-04 |  |
|  | 20% WRB sediment / 80% sand | 13 | 1.6E-01 | 2.9E-03 | 1.1E-06 |
| Soil depth | 5 cm | 17 | 9.5E-02 | 5.9E-03 |  |
|  | 4 cm | 14 | 7.6E-02 | 5.3E-03 | 4.7E-01 |
| soil depth 50% WRB Sediment / 50% sand | 5 cm | 10 | 4.1E-02 | 1.5E-04 |  |
|  | 4 cm | 8 | 2.0E-02 | 2.1E-06 | 5.0E-04 |
| soil depth 20% WRB Sediment / 80% sand | 5 cm | 7 | 1.7E-01 | 3.5E-03 |  |
|  | 4 cm | 6 | 1.5E-01 | 2.4E-03 | 4.6E-01 |

Table A5: Calculation of water residence time through the media using results of hydraulics conductivity test with constant head of both 50% WRB sediment and 50% sand and 20% WRB sediment / 80% sand. Extrapolation of data was used to calculate the residence time for sediment mix containing 40% WRB sediment / 60% sand and 75% WRB sediment / 25% sand.

| **Sediment mix** | **Sediment depth (cm)** | **Water depth (cm)** | **Average flow (L/min) (for 0.0079 m2)** | **Flow rate (m³/s)** | **Residence time (s)** |
| --- | --- | --- | --- | --- | --- |
| 50% WRB sediment and 50% sand | 5 | 2 | 0.0596 | 9.93E-07 | 159 |
|  |  | 1 | 0.0524 | 8.73E-07 | 90 |
|  | 4 | 2 | 0.0501 | 8.35E-07 | 189 |
|  |  | 1 | 0.0218 | 3.63E-07 | 217 |
| 20% WRB sediment / 80% sand | 5 | 2 | 0.237 | 3.95E-06 | 40 |
|  |  | 1 | 0.222 | 3.70E-06 | 21 |
|  | 4 | 2 | 0.0596 | 4.13E-06 | 38 |
|  |  | 1 | 0.0524 | 3.82E-06 | 21 |
| 40% WRB sediment / 60% sand | 5 | 2 | 0.1188 | 1.98E-06 | 119 |
|  |  | 1 | 0.1090 | 1.82E-06 | 67 |
| 75% WRB sediment / 25% sand | 5 | 2 | 0.0596 | 9.93E-07 | 218 |
|  |  | 1 | 0.0524 | 8.73E-07 | 147 |

Table A6: Results of benchtop seepage test.

| **Date** | **12-Jun** | | | | | | | | | | | |
| --- | --- | --- | --- | --- | --- | --- | --- | --- | --- | --- | --- | --- |
| **Water** | **Deionised water** | | | | | | **Synthetic Stormwater** | | | | | |
| **Experiment** | **Exp A** | | | **Exp B** | | | **Exp A** | | | **Exp B** | | |
| **Collection Time/ Compound** | **NO_3_-N (mg/L)** | **DRP (mg/L)** | **Mass (g)** | **NO_3_-N (mg/L)** | **DRP (mg/L)** | **Mass (g)** | **NO_3_-N (mg/L)** | **DRP (mg/L)** | **Mass (g)** | **NO_3_-N (mg/L)** | **DRP (mg/L)** | **Mass (g)** |
| **Wash Off** |  |  |  |  |  |  | 0.371 | 0.122 | 380 | 0.310 | 0.152 | 320 |
| **T0-T15** | 4.458 | 0.117 | 325 | 3.200 | 0.100 | 564 | 0.438 | 0.158 | 218 | 0.352 | 0.185 | 234 |
| **T15-T30** | 1.165 | 0.114 | 275 | 0.562 | 0.105 | 224 | 0.441 | 0.188 | 193 | 0.349 | 0.200 | 187 |
| **T30-T45** | 0.650 | 0.113 | 212 | 0.455 | 0.130 | 232 | 0.423 | 0.186 | 194 | 0.348 | 0.213 | 196 |
| **T45-T60** | 0.372 | 0.115 | 234 | 0.241 | 0.141 | 198 | 0.413 | 0.206 | 209 | 0.266 | 0.213 | 212 |
| **Blank** | 0.008 | n.d. | n.a. | n.a. | n.a. | n.a. | 0.407 | 0.227 | n.a. | n.a. | n.a. | n.a. |
| **Date** | **14-Jun** | | | | | | | | | | | |
| **Wash Off** | 0.273 | 0.195 | 850 | 0.055 | 0.144 | 875 | 0.256 | 0.171 | Not measured | 0.222 | 0.196 | Not measured |
| **T0-T15** | 0.129 | 0.213 | 218 | 0.027 | 0.194 | 276 | 0.315 | 0.202 |  | 0.259 | 0.231 |  |
| **T15-T30** | 0.089 | 0.211 | 146 | 0.015 | 0.245 | 197 | 0.337 | 0.236 |  | 0.331 | 0.250 |  |
| **T30-T45** | 0.064 | 0.228 | 146 | 0.009 | 0.288 | 192 | 0.359 | 0.233 |  | 0.352 | 0.254 |  |
| **T45-T60** | 0.045 | 0.251 | 130 | 0.008 | 0.301 | 163 | 0.372 | 0.239 |  | 0.366 | 0.251 |  |
| **Blank** | n.a. | n.a. | n.a. | 0.009 | n.d. | n.a. | n.a. | n.a. |  | 0.406 | 0.215 |  |
| **Date** | **15-Jun** | | | | | | | | | | | |
| **Wash Off** | 0.106 | 0.179 | 760 | 0.088 | 0.151 | 820 | 0.281 | 0.202 | 475 | 0.294 | 0.210 | 627 |
| **T0-T15** | 0.033 | 0.244 | 217 | 0.028 | 0.159 | 358 | 0.319 | 0.248 | 170 | 0.311 | 0.245 | 139 |
| **T15-T30** | 0.027 | 0.304 | 139 | 0.018 | 0.200 | 148 | 0.384 | 0.187 | 140 | 0.349 | 0.211 | 131 |
| **T30-T45** | 0.021 | 0.335 | 117 | 0.017 | 0.227 | 128 | 0.393 | 0.198 | 131 | 0.355 | 0.227 | 117 |
| **T45-T60** | 0.014 | 0.234 | 143 | 0.016 | 0.348 | 126 | 0.396 | 0.202 | 132 | 0.369 | 0.217 | 137 |
| **Blank** | 0.009 | n.d. | n.a. | n.a. | n.a. | n.a. | 0.406 | 0.213 | n.a. | n.a. | n.a. | n.a. |

Note: values of NO_3_-N lower than 0.045 mg/L are highlighted in red and it is considered below detection limits due to analysis method; n.d. = not detected or below detection limits; mass of experiment A and B containing SSW performed on 12-Jun did not have the mass measured; wash off of experiments A and B performed on 12-Jun containing deionised water were not analysed due to large amount of bed sediment washed off.
